# Modeling a New Water Channel That Allows SET9 to Dimethylate p53

**DOI:** 10.1371/journal.pone.0019856

**Published:** 2011-05-19

**Authors:** Qifeng Bai, Yulin Shen, Xiaojun Yao, Fang Wang, Yuping Du, Qin Wang, Nengzhi Jin, Jun Hai, Tiejun Hu, Jinbo Yang

**Affiliations:** 1 School of Life Science, Lanzhou University, Lanzhou, Gansu, China; 2 Gansu Computing Center, Lanzhou, Gansu, China; 3 School of Chemistry, Lanzhou University, Lanzhou, Gansu, China; 4 Department of Molecular Genetics, Lerner Research Institute, The Cleveland Clinic, Cleveland, Ohio, United States of America; Kings College, United Kingdom

## Abstract

SET9, a protein lysine methyltransferase, has been thought to be capable of transferring only one methyl group to target lysine residues. However, some reports have pointed out that SET9 can dimethylate Lys372 of p53 (p53-K372) and Lys4 of histone H3 (H3-K4). In order to understand how p53 can be dimethylated by SET9, we measured the radius of the channel that surrounds p53-K372, first on the basis of the crystal structure of SET9, and we show that the channel is not suitable for water movement. Second, molecular dynamic (MD) simulations were carried out for 204 ns on the crystal structure of SET9. The results show that water leaves the active site of SET9 through a new channel, which is made of G292, A295, Y305 and Y335. In addition, the results of molecular docking and MD simulations indicate that the new water channel continues to remain open when S-adenosyl-L-methionine (AdoMet) or S-adenosyl-L-homocysteine (AdoHcy) is bound to SET9. The changes in the radii of these two channels were measured in the equilibrium phase at the constant temperature of 300 K. The results indicate that the first channel still does not allow water to get into or out of the active site, but the new channel is large enough to allow this water to circulate. Our results indicate that water can be removed from the active site, an essential process for allowing the dimethylation reaction to occur.

## Introduction

Mono-, di- and tri-methylations of the lysine residues of proteins play an important role in gene regulation. They not only regulate gene expression and the differentiation of embryonic stem cells [1], [2] but also have a potential therapeutic benefit in cancer [3], [4]. SET9, also called SET7 and SET7/9, was purified in 2002 [5], [6]. It is one of the protein lysine methyltransferases (PKMTs) and is responsible for pivotal reactions in the regulation of chromatin structure. SET9 was reported to methylate histone H3 at Lys4 (H3-K4) *in vivo* [7] and p53 at Lys372 (p53-K372) *in vitro* [8]. p53 is a tumor suppressor helps to regulate many cellular functions, such as apoptosis, DNA repair, the cell cycle, and senescence [9].

The crystal structure of SET9 in complex with S-adenosyl-L-homocysteine (AdoHcy) and H3-K4 shows that it should only transfer one methyl group to H3-K4, because water occupies the position that would need to be empty to accomodate a second methyl group [10]. The monomethylated Lys372 residue of p53 (p53-K372Me1) is also similarly situated in the crystal structure of SET9 [8]. However, in contrast to the these conclusions results, some published evidence supports the idea that SET9 is able to dimethylate H3-K4 and p53-K372 when the unmodified substrate is bound to the active site of the enzyme [11], [12]. Furthermore, Yang *et al*. [13] reported that STAT3 is dimethylated on K140 by SET9 when it is bound to a subset of the promoters that it activates. This methylation of K140 is a negative regulatory event, because its blockade greatly increases the steady-state amount of activated STAT3 and the expression of a subset of STAT3 target genes [13]. Chakrabarti *et al*. [14] and Francis *et al*. [15] have previously shown that the insulin promoter exhibits a high degree of H3-K4 dimethylation in B-cells and that this dimethylation is largely dependent upon the recruitment of SET9 to the promoter. Deering *et al*. [16] revealed important roles for SET9 as a dimethyltransferase in regulating the transcription of the insulin gene as well as other important B-cell genes. Three genes - Ins1/2, Mafa, and Glut2 (Slc2a2) - were consistently down-regulated when SET9 was depleted. Reduction of Ins1/2 and Glut2 mRNAs was also found to be associated with reduced H3-K4 dimethylation in the promoter region of these two genes.

The mechanism of p53 monomethylation has been illustrated through computer simulations. It is believed that water plays an important role and its presence at the active site of SET9 is considered to favor the transfer of only one methyl group to the N_ζ_ atom of p53-K372, disrupting the formation of conformations necessary to carry out the dimethylation reaction [17]–[20]. However, these important computational models did not answer a crucial question: can water leave the active site of SET9 through a channel following the monomethylation reaction? If so, SET9 might be able to dimethylate p53, since water takes up the position of the second methyl group at the active site.

In addition, dimethylation has recently been revealed by real experiments when the key water is removed from the active site of SET9. It was found that other waters formed CH…O hydrogen bonds with the methyl group recognized by SET domain of lysine methyltransferases [21]. However, the key water of SET9 formed an NH…O hydrogen bond. So other waters do not play a major role in hydrating methyl groups. Furthermore, Y295 of SET9 can form CH…O hydrogen bond with substrate for the dimethylation reaction when water dissociated from the active site, leaving space for the methyl group to shift and project into the solvent pocket [22]. But these real experiments have not shown which water channel is chosen for the key step of allowing water to flow out of the active site of SET9.

In the present work, we measured the radii of the first possible channel and the p53-K372 in SET9. Compared with the radius of water, the results indicate that this channel is not suitable for water movement. We then performed molecular dynamics (MD) simulations on the crystal structure of SET9 with explicit solvent at constant pressure and constant temperature, with time steps of 1 fs. The results show that water leaves the active site for a period of time through the new channel which is surrounded by G292, A295, Y305 and Y335 of SET9. This new channel keeps its open state even when S-adenosyl-L-methionine (AdoMet) or AdoHcy binds to the cavity of SET9. Furthermore, the radius changes of the first channel and the new water channel were also measured at constant temperature when the whole system was at equilibrium. The results suggest that the new channel is suitable for water movement and that the water flow through the first channel is still prohibited. The current study indicates that water can be removed through the new water channel as a necessary condition of the dimethylation reaction.

## Results and Discussion

### Structural features of SET9 and channel radii

**Figure 1:**
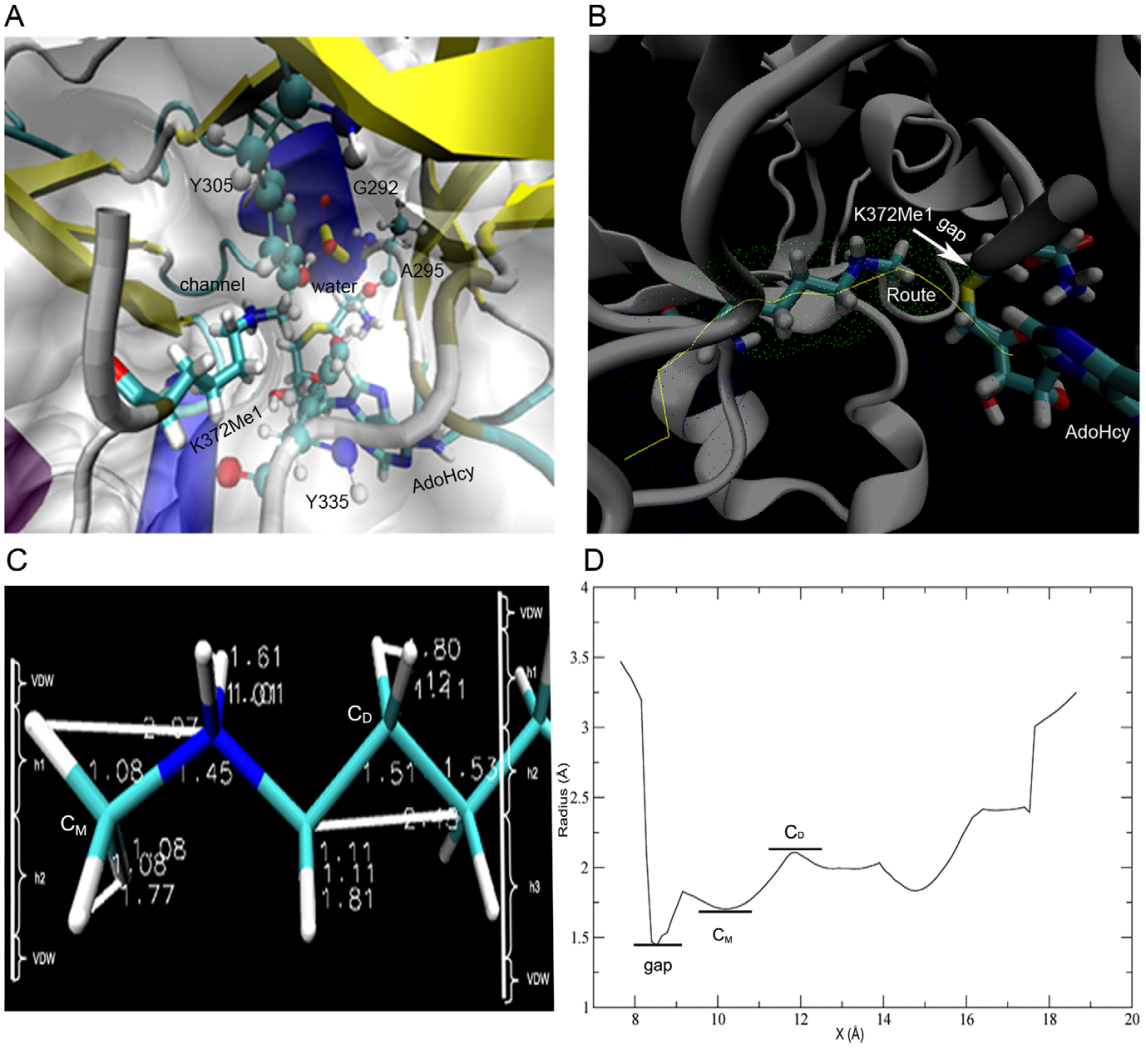
Structure features of SET9 and calculation of channel radii. (A) The general crystal structure of SET9 is displayed. The K372Me1 residue of p53 (p53-K372Me1) inserts into the channel of SET9. The water near p53-K372Me1 in the channel is colored yellow. This water forms three hydrogen bonds with G292, A295 and Y305 at the active site. (B) The first explored channel of SET9 is shown. The yellow line is the route of the channel. The grid points represent the surface of the channel. The arrow represents the gap spaces which have no substrate. (C) Assessment of the geometry radii of the major structure of p53-K372. *h1*, *h2* and *h3* are the corresponding height of molecular shape, and VDW is the van der Waals radius of the atom. C_D_ and C_M_ are the third and last carbons of side-chain group of p53-K372. (D) Radii of channels along the *x* axis. Gap represents the area between the p53-K372Me1 and AdoHcy as Figure 1B. C_D_ and C_M_ show the area of the third and last carbons of side-chain group of p53-K372 in the first channel.

The general structure and important active sites in SET9 are depicted in Figure 1A. A glance at one side of SET9 reveals that the p53 peptide inserts into the channel in which the mono- or dimethylated reaction occurs. AdoHcy binds to the opposite cavity of p53-K372Me1 residue. Only one water molecule is close to the N_ζ_ atom of p53-K372Me1 in the complex structure, but it anchors in the active site of SET9. This key water, which forms hydrogen bonds with G292, A295 and Y305, occupies the position of the potential second methyl group of p53-K372Me1. We also compared the crystal structures of SET9 in complex with H3 and p53, and found that the key water takes up the same position in SET9 (Figure S1). Therefore, it is important to find a potential channel that would allow the water to escape.

The channel formed for the methylation of p53 represents one possibility. Exploring the radii of such channels in SET9 is fundamental for this study, as the aim of the current work is to determine whether the water is allowed to pass through this channel or not. Figure 1B shows that the route is along the direction of the channel. If the water leaves the active site along this route, it would be blocked from leaving the channel by p53-K372Me1 and AdoHcy. Figure 1C shows the main structure and some bond lengths of p53-K372 (Table S1). According the major geometry of substrate molecule, the radius *R(j)* of substrate cross-section can be evaluated as
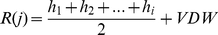
(1)


Where *h_i_* is approximately equal to the height of the triangle formed by three atoms. The calculation method for *h_i_* is shown in Formulas S1. VDW is the van der Waals radius of each atom. The radius calculation takes into account the VDW radii because two molecules should keep out of this distance under normal circumstances (Formulas S2). Two radii were measured, one in the C_M_ area at the end methyl of side-chain group of p53-K372, another in the C_D_ area at the third carbon atom of side-chain group of p53-K372. As marked in Figure 1D, C_M_ is in the lower radius area, which is at the end of p53-K372. The C_D_ area has a larger radius than other places which are occupied by the substrate. By computing the areas of C_M_ and C_D_, the radius range of the substrate in the channel is estimated to be from 1.76 to 2.19 Å, and the channel radius of the original crystal structure is 1.87 Å by computing the average of radii between 8.403 and 15.403 Å along the *x* axis in Figure 1D. The smaller radius is lower than 1.5 Å in the gap area (Figure 1B and D). AdoHcy also blocks the pore at one side, so water can't enter the channel from this side. On the other side, p53-K372 occupies most of the channel space. Compared with the channel radii, it seems that the substrate is tightly surrounded by this channel. By subtracting the smallest radius of the substrate from the channel radii, we see that the water molecule cannot leave or enter the active site of SET9 because water can occupy a radial sphere of 1.9 Å if the protein interacts with the water [23]. Therefore, this channel can be excluded for water movement preliminarily.

### The water leaves the active site from the new channel for a period of time

It is necessary to study how water flows at the active site, since the position of water is so important that it could determine whether SET9 can methylate p53-K372Me1 or not. This water locates at the active site through three hydrogen bonds with G292, A295 and Y305. G292 and A295 are as hydrogen bond acceptors while Y305 serves as hydrogen bond donor. Because these residues form a dynamic steady conformation, once the water gets into the active site, it will occupy the position of the potential second methyl group of p53-K372Me1.

**Figure 2:**
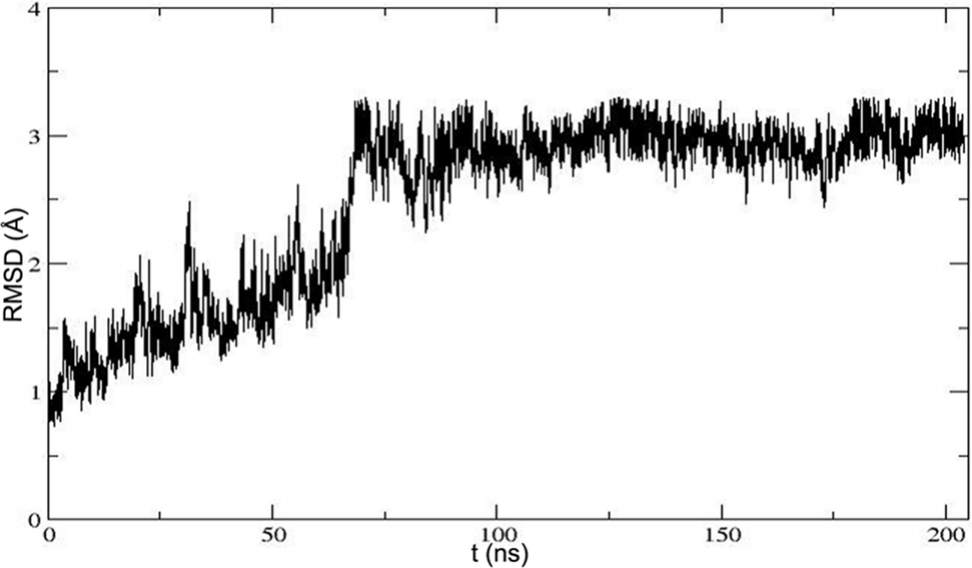
Time evolution of RMSD over the backbone atoms forming SET9 with respect to the crystallographic structure.

To find the new channel for water movement, MD simulations were carried out on the crystal structure of SET9 in complex with AdoHcy and the p53 peptide in an explicit solvent. The developed force field parameterizations for p53-K372Me1 are listed in File S1. A primary analysis of MD simulation involves the root mean square deviation (RMSD) profile for the protein backbone atoms with respect to the crystal structure after equilibrating 1 ns simulation, in order to find the equilibration phase for studying the water at the active site of SET9 in the model during the simulation (Figure 2). The value of RMSD, which is about 3 Å after 70 ns, is slightly high because SET9 contains some disordered and flexible sequences such as residues 186–209, 332–350. So the structure of SET9 is distorted normally during the MD simulation. A similar phenomenon has been reported previously [24], [25].

**Figure 3:**
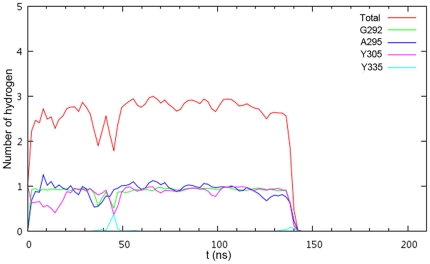
Hydrogen bonds between the water and four residues changed with simulated time. The red line represents the total number of hydrogen bonds.

**Figure 4:**
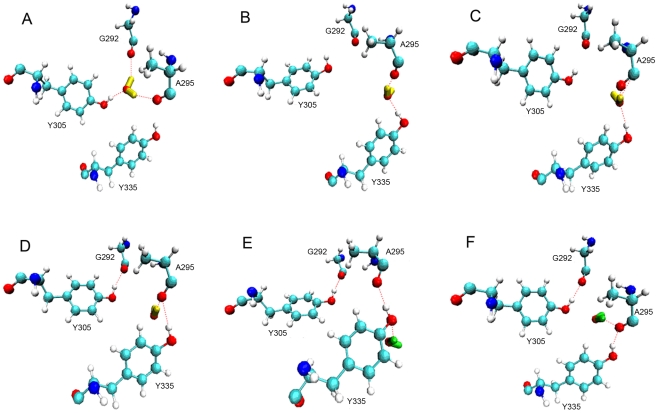
The detail of water flow. (A) Most of the conformation in this MD simulation. (B) At time t = 44.120 ns; (C) at t = 138.680 ns, and the linking line between hydrogen of water and carbon atom of A295 does not represent the hydrogen bond. It depicts more recent geometric distance than in Figure 4B; (D) at t = 139.200 ns; (E) at t = 146.640 ns; (F) at t = 146.642 ns. The water colored yellow is a crystal water of SET9 while the green one is recruited from the water box.

For the detailed behavior of the water, the long MD simulation was carried out in a total time of 204 ns. The number of hydrogen bonds between water and G292, A295, Y305, Y335 residues was calculated as a function of time for this long MD simulation (Figure 3). There are six states in the water flowing process (Figure 4). The water forms three hydrogen bonds with the three residues G292, A295 and Y305 most of the time (Figure 4A). At the same time, some special conformations are observed at different times. At t = 44.120 ns, hydrogen and oxygen of water form a hydrogen bond with the oxygen of A295 and hydrogen of Y335, respectively (Figure 4B). This condition is reflected at about 45 ns in the Figure 3: The number of hydrogen bonds increases for Y335. It does not change the tendency for A295, but it decreases for G292 and Y305 at the same time. However, this conformation isn't close enough to the carbon atom of A295, so it is insufficient to allow the water to move away through this new channel. Lately, the water recovers the state as Figure 4A. At t = 138.680 ns, the system is in the equilibration phase as shown in Figure 2. Meanwhile, the water in Figure 4C is closer to A295 than in Figure 4B. In succession, there is only one hydrogen bond between the water and A295 (Figure 4D). This condition corresponds to Figure 3 at about 140 ns: the number of hydrogen bonds for G292 and Y305 are reduced quickly, and then the tendency of hydrogen bond numbers for A295 is also downward after a transient time. In addition, the water forms one hydrogen bond with Y335, so there is a peak between 130 ns and 140 ns in Figure 3. Finally, the water is released quickly from the active site of SET9. At t = 146.640 ns, the new water is captured by Y335 (Figure 4E). Subsequently, this new water, which is close to the center of G292, A295 and Y305 (Figure 4F), is passed to Y295. At last, it is easy to form a stable conformation at the active site of SET9 as in Figure 4A (Movie S1). It is obvious that Y335 plays a water-outlet role as well as a water-trapping role in this dynamic flowing course.

**Figure 5:**
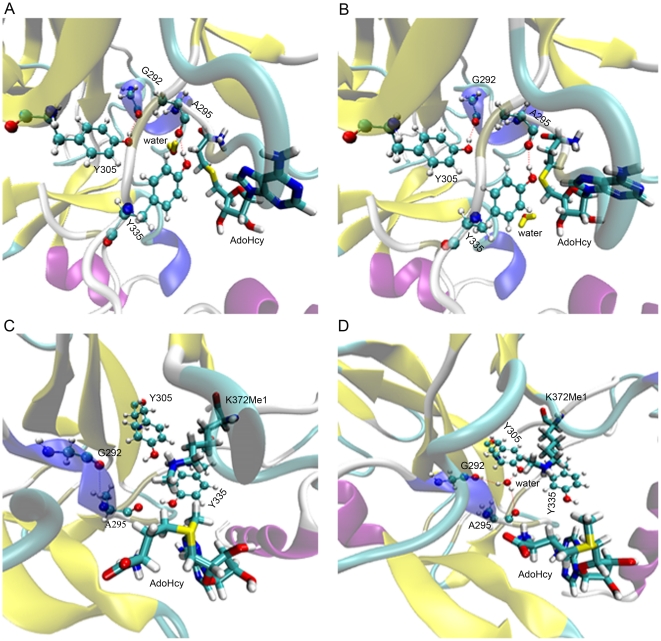
Molecular docking and MD simulations. (A) The water is leaving in the channel at t = 139.200 ns. (B) The water has left the channel along the new channel at t = 139.400 ns. (C) The conformation of the AdoMet binding to SET9 after molecular docking. (D) One water molecule enters into the active site of SET9 during the MD simulations.

As an overview of these MD simulations, water can leave the active site through a new channel (Figure 5A and B). This channel, which only contains one water molecule, is formed by the G292, A295, Y305, and Y335 residues of SET9. At the same time, it is noteworthy that there is no water at the active site of SET9 for about 7.44 ns, suggesting that the key water can be removed from the active site of SET9 for a period time.

### New water channel keeps opening and water can pass through this new channel

AdoHcy is the derivative metabolite of the cofactor AdoMet. Both AdoHcy and AdoMet can bind to the cavity of SET9 near the new water channel. AdoMet has one more methyl group than AdoHcy (Figure S2). The flexible docking models of AdoMet and AdoHcy were carried out on the binding cavity of SET9 model structure which was cut out from the 16,000th frame of 204 ns MD simulations. The docking energy score of AdoHcy is −65.93 kcal/mol, while for the binding energy of AdoMet, it is −68.62 kcal/mol. Based on the binding pose of AdoMet (Figure 5C), the methyl group of AdoMet was observed to enhance the binding ability of AdoMet in the groove of SET9. In order to explore whether the methyl group of AdoMet can block the water channel, MD simulations were performed on a model structure with no water at the active site of SET9. After MD simulations, we found that the water could enter the active site of SET9 (Figure 5D), suggesting that the new water channel has not been shut down by the methyl group of AdoMet. The new water channel allows water to flow at the active site regardless of whether AdoMet or AdoHcy is bound to the cavity region of SET9.

**Figure 6:**
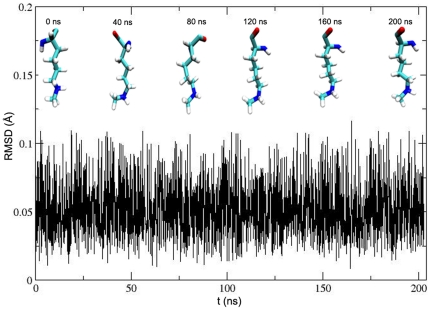
RMSD of the crystallographic p53-K372 backbone atoms with respect to simulated conformation as a function of time.

In order to get the radius changes of the first impossible water channel and the new water channel at a constant temperature of 300 K in the equilibrium phase, the frames, which are collected from 140 ns, 150 ns, 160 ns, 170 ns and 180 ns trajectory file, are used as models for the radius measurement. As shown in Table 1, the minimal radius of the first channel is not more than 1.96 Å. So it is not easy for water to move in the channel. In addition, the pore of the first channel is also blocked by AdoHcy. The sizes of the first channel radius are about 0.3 Å larger than the sizes of the crystal structure channel radius when the whole system is in the equilibrium phase at the constant temperature of 300 K. At the same time, the RMSD profile shows that the conformation of p53-K372 is not distorted hugely with simulation time (Figure 6). The snapshots every 40 ns in Figure 6 also show that the major conformation of p53-K372 changes slightly. So the radii, which are computed as in Figure 1C, can still be used to estimate the occupied space of p53-K372 roughly. The channel radii only enlarge by about 0.3 Å, but the substrate still takes up the minimal space of radius of 1.76 Å. The remaining radius is about 0.7 Å, though the minimal radius is subtracted from the highest radius of C_D_ area of the first channel. So it is believed that the water still cannot enter or leave the active site from the first channel. For the radii of new water channel (Table 1), the minimal radii are slightly larger than water radius. The average radius is about 2.3 Å which is large enough for water movement. There is no substrate to block the pore of the water channel as in the simulated results before. So the water can enter or leave the active site from this new channel. If the water is removed from this new channel, the dimethylation reaction can be carried out, as real experiments have reported [21], [22].

**Table 1:** The minimal and average radii calculated at different simulated time when the whole system is in the equilibrium phase.

Time (ns)	R_Fmin_ a(Å)	R_Favg_ b(Å)	R_Nmin_ c(Å)	R_Navg_ d(Å)
140	1.80	2.15	1.96	2.29
150	1.86	2.21	2.01	2.23
160	1.73	2.08	2.11	2.36
170	1.76	2.06	1.98	2.21
180	1.87	2.18	2.08	2.28

a Minimal radius of the first channel.

b Average radius of the first channel.

c Minimal radius of the new water channel.

d Average radius of the new water channel.

In conclusion, we demonstrate evidence for a new channel that lets water flow in and out of the active site of SET9. The channel and p53-K372 radii were measured to determine whether the water is able to pass through the first potential channel. The result excluded this channel, and indicated that there was another new channel for water movement. During the equilibration phase of the MD simulation, we observed that the water circulated through the new channel which still stayed open with AdoHcy or AdoMet bound. In addition, the radius changes of the first impossible channel and the new water channel at constant temperature prove that the water will choose the new channel for circulation. It is reasonable to believe that there exists the same new water channel for SET9 with histone H3, since the substrate regions of H3 and p53 have similar conformations and the second methyl positions are both occupied by the water at the active site of SET9.

## Materials and Methods

### Construction of the starting molecular model and molecular force field parameterizations

The molecular model 1XQH [8] is made of a dimer (subunits A and B) and 717 water molecules from Protein Data Bank (PDB). Each subunit of 1XQH contains the crystal structure of SET9, the six-peptide substrate of p53 and S-adenosyl-L-homocysteine (AdoHcy). The subunit A of 1XQH was chosen as a starting structure for exploring the radii and carrying out the molecular dynamics (MD) simulations.

The parameterizations of monomethylated Lys372 residue of p53 (p53-K372Me1) which was not found in CHARMM27 force field (version 31) [26] were developed: the geometry optimization uses RHF/6–31G* model with tight SCF convergence criteria; for single point calculation, it was computed at the RHF/6–31G* level of theory with tight SCF convergence criteria, and all orbital symmetry constraints were lifted. The topology and parameters for AdoHcy and S-adenosyl-L-methionine (AdoMet) were extracted from the stream file of CHARMM27 force field (version 31) which patches to create model compounds.

The force field parameterizations were developed by using VMD Paratool Plugin v1.2 [27] and Gaussian98 Revision A.9 [28] on the 8 cores of an array of two 2.33-GHz Intel(R) Xeon(R) processors.

### Radius measurement

As an overview of the starting structure, the space which surrounds p53 peptide is a potential channel for water flow. To measure the radius of this channel, AdoHcy and p53 peptide were deleted from the starting structure. The exploring vector was along the *x* axis. And the average coordinate of C_α_ and N_ζ_ atom of p53-K372 was used as an initial point for exploring the whole channel. The maximum value of radius for the end of the pore was 3.5 Å. While for the value of the distance between the planes, it was set as 0.25 Å. The VDW radius of hydrogen is 1.09 Å.

For the radii of p53-K372, the length between two different atoms is measured by VMD 1.8.6 [29]. The complex of SET9 was dealt by using UCSF Chimera 1.4.1 [30] and the radii of channel were measured by performing HOLE v2.2 program [31].

### MD simulations and molecular docking

The model structure of the SET9, p53 peptide, crystal waters and AdoHcy was rebuilt on the basis of CHARMM27 force field (version 31). An empirical water model, TIP3P [32], was used to construct the water box which dimensions were 100 × 90 ×75 Å^3^. MD runs at a constant temperature of 300 K and pressure of 1 bar, employing the Langevin piston [33] and softly damped Langevin dynamics with a time setup of 1 fs. And the particle-mesh Ewald method [34] was used to calculate electrostatic interactions. The MD simulations were carried out in three steps: minimization period of 0.5 ns, equilibration period of 1 ns and product simulation period of 204 ns. The minimization uses a conjugate gradient method and the equilibration was performed with a damping coefficient of 1/ps for Langevin dynamics at 300 K. Only the frames reserved during the product simulation were considered, with a frame stored each 10,000 iterations (10.0 ps), yielding 20,400 frames. In preparation of a statistical number of hydrogen bonds, the distance between the donor and acceptor atoms were defined as less than 3.5 Å and the angle was set less than 35° [35], [36]. The radii of different frames were also calculated by the same parameters as the first radius measurement.

When the first MD simulations were finished, the second MD simulations continued to be carried out. The model structure of SET9 was taken from the 16,000th frame which is in the equilibrium phase of 204 ns MD simulations (Protocol S1). The structures of AdoMet (Compound ID: 34756) and AdoHcy (Compound ID: 439155) were retrieved from the PUBCHEM. All waters were deleted from the model structure. The binding cavity of AdoHcy on SET9 was the region targeted for docking. The docking structure of SET9 in complex with AdoMet and K372Me1 was used for further MD simulation. In order to get effective evidence, the docking structure model was built and calculated with the same parameters as the starting structure. The MD simulation was also carried out in three steps: minimization period of 0.5 ns, equilibration period of 1 ns and product simulation period of 23 ns. There only the frames reserved during the product simulation were considered, with a frame stored each 1000 iterations (1.0 ps), yielding 23000 frames.

The program UCSF DOCK (Version 6.0) [37] was used to perform the molecular docking with a flexible algorithm. Molecule preparation and trajectory analysis were done by using VMD 1.8.6 [29]. Display and characterization of chemical structures were performed by using Marvin 5.2.2 [38]. The MD simulation runs on the 100 cores of twenty-five 2218-Hz AMD Opteron(tm) processors by using NAMD 2.7b2 [39] in PC clusters.

## Supporting Information

Figure S1 The crystal structures of SET9 in complex with H3 and p53. (A) The complex of SET9 and H3 peptides. The water colored red forms hydrogen bonds with Y305, G292 and A295. This figure was made by 1O9S file which was extracted from Protein Data Bank (PDB). (B) The complex of SET9 and p53 peptides. The water colored blue forms the same hydrogen bonds with the water as Figure S1A.
(TIF)

Figure S2 The structures of AdoHcy and AdoMet.
(TIF)

Formulas S1 (DOC)

Formulas S2 (DOC)

Movie S1 (MPG)

File S1 (DOC)

Protocol S1 (DOC)

Table S1 The distances between atoms of p53-K372
(DOC)
