# Supplementary figures and images for: Modeling a New Water Channel That Allows SET9 to Dimethylate p53
Source: PLoS One. 2011 May 19;6(5):e19856. doi: 10.1371/journal.pone.0019856 (PMC3098259; doi:10.1371/journal.pone.0019856)

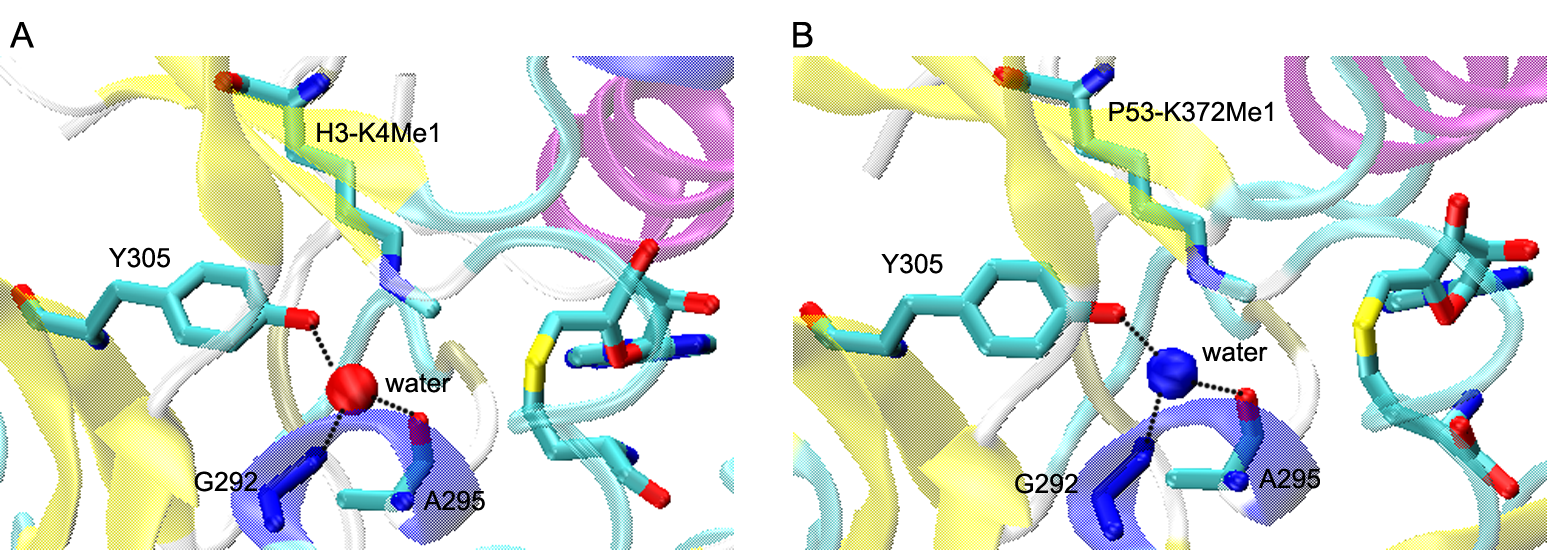

Supplement: Figure S1 — The crystal structures of SET9 in complex with H3 and p53. (A) The complex of SET9 and H3 peptides. The water colored red forms hydrogen bonds with Y305, G292 and A295. This figure was made by 1O9S file which was extracted from Protein Data Bank (PDB). (B) The complex of SET9 and p53 peptides. The water colored blue forms the same hydrogen bonds with the water as Figure S1A. (TIF) [file pone.0019856.s001.tif]

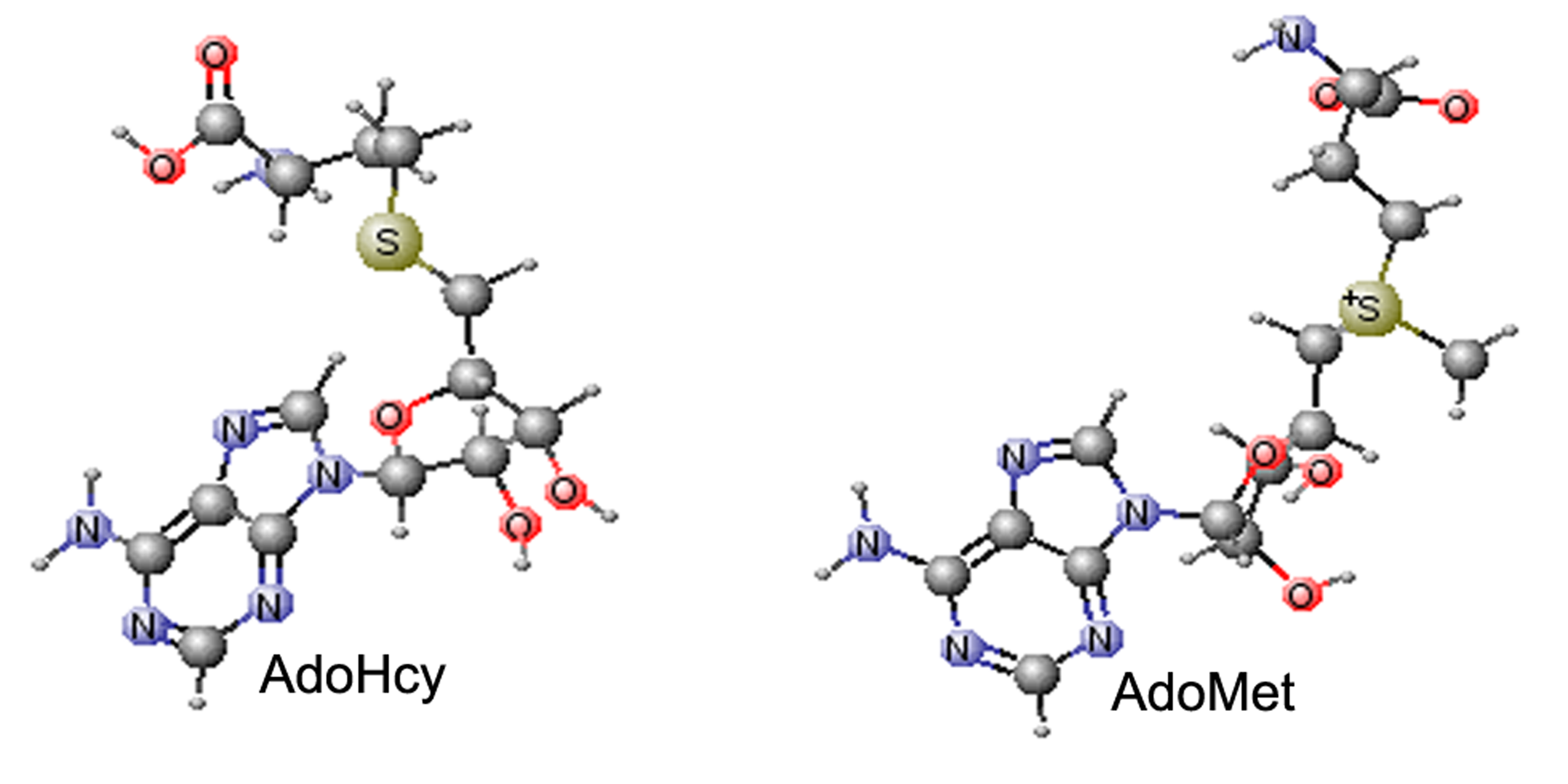

Supplement: Figure S2 — The structures of AdoHcy and AdoMet. (TIF) [file pone.0019856.s002.tif]
