# Supplementary material for: Modeling a New Water Channel That Allows SET9 to Dimethylate p53
Source: PLoS One. 2011 May 19;6(5):e19856. doi: 10.1371/journal.pone.0019856 (PMC3098259; doi:10.1371/journal.pone.0019856)
Supplement: Formulas S1 — (DOC) [file pone.0019856.s003.doc]

***The calculation of the triangle height***

According the cosine rule, it has:

$\cos\theta=(a^{2}+b^{2}+c^{^{2}})/2ab$ (1)

Where a, b and c are represented the three sides of the triangle. So the area of triangle is:

$\begin{matrix} S=1/2*ab*\sin C \\ =1/2*\sqrt{1-\cos\theta} \\ =1/2*\sqrt{1-(a^{2}+b^{2}+c^{2})^{2}/4a^{2}b^{2}} \\ =1/4*\sqrt{(a+b+c)(a+b-c)(b+c-a)(a+c-b)} \end{matrix}$ (2)

The area of triangle also equals to:

$S=1/2*ah$ (3)

Where *h* is the height of the triangle.

Integrating the equation (2) and (3), we can get *h*:

$h=\frac{1}{2a}\sqrt{(a+b+c)(a+b-c)(a+c-b)(b+c-a)}$ (4)
