# Supplementary material for: Modeling a New Water Channel That Allows SET9 to Dimethylate p53
Source: PLoS One. 2011 May 19;6(5):e19856. doi: 10.1371/journal.pone.0019856 (PMC3098259; doi:10.1371/journal.pone.0019856)
Supplement: Formulas S2 — (DOC) [file pone.0019856.s004.doc]

***The calculation of radius***

The radii of hole are evaluated through many spheres. The radius *R*(*p*) of every sphere can be computed without overlapping any atom:

$$R(p)=\min_{i=1}^{N_{atom}} [|x_{i}-p|-vdW_{i}]$$

In this formula, it is obviously that the van der Walls (VDW) radius of atom is subtracted when the radii of channel are evaluated by Hole program. The reason is that two molecules do not keep in touch normally in the range of VDW radii. So when we estimate the radii of substrate (p53-K372), we should consider the VDW radii. This consideration is not only consistent with the algorithm of the Hole program, but also the VDW radii contribute the enough length in the channel.
