# Supplementary material for: Modeling a New Water Channel That Allows SET9 to Dimethylate p53
Source: PLoS One. 2011 May 19;6(5):e19856. doi: 10.1371/journal.pone.0019856 (PMC3098259; doi:10.1371/journal.pone.0019856)
Supplement: File S1 — (DOC) [file pone.0019856.s006.doc]

!====================================================================================

! CHARMM topology for methylated p53-K372

!====================================================================================

! Mass entry

!----------------------------------------------------------------------------------------------

MASS 1 CNT2 12.01100 C !

MASS 2 CT2 12.01100 C !

MASS 4 HA 1.00800 H !

MASS 5 HN2 1.00800 H !

MASS 6 HNA2 1.00800 H !

MASS 7 N2 14.00700 N !

!---------------------------------------------------------------------------------------------

! Residue

!----------------------------------------------------------------------------------------------

RESI MLZ 1.00 ! Methylated p53-K372

GROUP ! Built by Gaussian 98 and VMD-PARATOOL

ATOM N NH1 -0.47 ! |

ATOM HN H 0.31 ! HN-N

ATOM CA CT1 0.07 ! | HB1 HG1 HD1 HE1 HZ1 HF1

ATOM HA HB 0.09 ! | | | | | / /

GROUP ! HA-CA--CB--CG--CD--CE--NZ---CF-HF2

ATOM CB CT2 -0.18 ! | | | | | \ \

ATOM HB1 HA 0.09 ! | HB2 HG2 HD2 HE2 HZ2 HF3

ATOM HB2 HA 0.09 ! O=C

GROUP ! |

ATOM CG CT2 -0.18

ATOM HG1 HA 0.09

ATOM HG2 HA 0.09

GROUP

ATOM CD CT2 -0.18

ATOM HD1 HA 0.09

ATOM HD2 HA 0.09

GROUP

ATOM CE CNT2 0.21

ATOM HE1 HNA2 0.05

ATOM HE2 HNA2 0.05

ATOM NZ N2 -0.32

ATOM HZ1 HN2 0.40

ATOM HZ2 HN2 0.40

ATOM CF CNT2 -0.06

ATOM HF1 HNA2 0.09

ATOM HF2 HNA2 0.09

ATOM HF3 HNA2 0.09

GROUP

ATOM C C 0.51

ATOM O O -0.51

BOND CB CA CG CB CD CG CE CD NZ CE CF NZ

BOND N HN N CA C CA

BOND C +N CA HA CB HB1 CB HB2 CG HG1

BOND CG HG2 CD HD1 CD HD2 CE HE1 CE HE2

DOUBLE O C

BOND NZ HZ1 NZ HZ2 CF HF1 CF HF2 CF HF3

IMPR N -C CA HN C CA +N O

CMAP -C N CA C N CA C +N

DONOR HN N

DONOR HZ1 NZ

DONOR HZ2 NZ

ACCEPTOR O C

!================================================================================

! CHARMM parameters for methylated p53-K372

!================================================================================

!BONDS

!------------------------------------------------------------------------------------

CT2 N2 252.154 1.5092

!--------------------------------------------------------------------------------

!ANGLES

CT2 CT2 N2 41.844 111.02

HA CT2 N2 16.335 106.35

HA CT2 N2 16.335 106.36

CT2 N2 HN2 13.738 108.86

CT2 N2 HN2 13.738 108.86

CT2 N2 CNT2 35.106 114.84

HN2 N2 HN2 5.341 105.43

!--------------------------------------------------------------------------------

!DIHEDRALS

CT2 CT2 N2 HN2 0.4390 3 0.00

CT2 CT2 N2 HN2 0.4390 3 0.00

CT2 CT2 N2 CNT2 0.4078 3 0.00

HA CT2 N2 HN2 0.4632 3 0.00

HA CT2 N2 HN2 0.4841 3 0.00

HA CT2 N2 CNT2 0.4399 3 0.00

HA CT2 N2 HN2 0.4841 3 0.00

HA CT2 N2 HN2 0.4632 3 0.00

HA CT2 N2 CNT2 0.4399 3 0.00

CT2 N2 CNT2 HNA2 0.4402 3 0.00

CT2 N2 CNT2 HNA2 0.4313 3 0.00

CT2 N2 CNT2 HNA2 0.4313 3 0.00
