# Supplementary material for: Modeling a New Water Channel That Allows SET9 to Dimethylate p53
Source: PLoS One. 2011 May 19;6(5):e19856. doi: 10.1371/journal.pone.0019856 (PMC3098259; doi:10.1371/journal.pone.0019856)
Supplement: Protocol S1 — (DOC) [file pone.0019856.s007.doc]

***Eextracting the 16000th frame from trajectory file***

(1) Loading the structure file of the protein, peptide, AdoHcy and waters, and then load the trajectory file.

(2). Using TCL script to saving the 16000th frame in VMD software as below:

************************************************************************

set pro [atomselect top protein frame 16000]

$pro writepdb SET9.pdb

************************************************************************

(3). Deleting the ligand (AdoHcy) of SET9 and dealing with the structure for docking by using UCSF Chimera.

(4). When the molecular docking was finished, the SET9 in complex with AdoMet and peptide can be rebuilt for MD simulations.
