# Supplementary material for: Modeling a New Water Channel That Allows SET9 to Dimethylate p53
Source: PLoS One. 2011 May 19;6(5):e19856. doi: 10.1371/journal.pone.0019856 (PMC3098259; doi:10.1371/journal.pone.0019856)
Supplement: Table S1 — The distances between atoms of p53-K372 (DOC) [file pone.0019856.s008.doc]

Table S1: The distances between atoms of p53-K372

| Distance between atoms | Distance value (Å) |
| --- | --- |
| CM-HCM1 | 1.08 |
| CM-HCM2 | 1.08 |
| CM-HCM3 | 1.08 |
| HCM3-Nξ | 2.07 |
| CM- Nξ | 1.45 |
| Nξ-HNξ1 | 1.01 |
| Nξ-HNξ2 | 1.01 |
| HNξ1-HNξ2 | 1.61 |
| CD-HCD1 | 1.11 |
| CD-HCD2 | 1.12 |
| HCD1-HCD2 | 1.80 |
| CD-CE | 1.51 |
| CD-CG | 1.53 |
| CE-CG | 2.48 |
| CE-HCE1 | 1.11 |
| CE-HCE2 | 1.11 |
| HCE1- HCE2 | 1.81 |

CM is the last carbon atom of side-chain group of p53-K372; HCM1, HCM2 andHCM3 are the hydrogen atoms on the CM atom.Nξ is the nitrogen atom of side-chain group of p53-K372. HNξ1 and HNξ2 are the hydrogen atoms on the Nξ atom. CD is the third carbon atom of side-chain group of p53-K372. HCD1 and HCD2 are the hydrogen atoms on the CD ­atom. CE is the forth carbon atom of side-chain group of p53-K372. HCE1 and HCE2 are the hydrogen atoms on the CE ­atom.
